# TJ0113-induced mitophagy in acute liver failure detected by Raman microspectroscopy

**DOI:** 10.1016/j.redox.2025.103654

**Published:** 2025-04-29

**Authors:** Chunlian Huang, Jiaqi Liao, Xufeng Cen, Changwei Jiao, Sijia Chen, Dong Liu, Hang-Shuai Qu, Jiansheng Zhu, Sailing He

**Affiliations:** a Department of Infectious Diseases, Taizhou Hospital of Zhejiang Province Affiliated to Wenzhou Medical University, Linhai, Zhejiang, 317000, China; b Centre for Optical and Electromagnetic Research, College of Optical Science and Engineering, Zhejiang University, Hangzhou, 310058, China; c MedEnglnfo Collaborative Research Center, Zhejiang Engineering Research Center for Intelligent Medical Imaging, Sensing and Non-invasive Rapid Testing, Taizhou Hospital, Zhejiang University, Taizhou, China; d National Engineering Research Center for Optical Instruments, Zhejiang University, Hangzhou, 310058, China; e Research Center of Clinical Pharmacy of the First Affiliated Hospital & Liangzhu Laboratory, Zhejiang University School of Medicine, Hangzhou, China; f Hangzhou PhecdaMed Co., Ltd. Third Floor, Building 2, No.2626. Yuhangtang Road, Yuhang District, Hangzhou City, Zhejiang Province, China; g Department of Public Laboratory, Taizhou Hospital of Zhejiang Province Affiliated to Wenzhou Medical University, Linhai, Zhejiang, 317000, China; h Department of Electromagnetic Engineering, School of Electrical Engineering, Royal Institute of Technology, 100 44, Stockholm, Sweden

**Keywords:** Raman signal, Point-scan Raman imaging, Aberration-free line-scanning confocal Raman imaging, Acute liver failure, Mitophagy, TJ0113

## Abstract

Impaired mitophagy underlies the pathophysiology of acute liver failure (ALF) and is closely associated with tissue damage and dysfunction. A novel mitophagy inducer, TJ0113, was used for treatment during ALF pathogenesis. In this study, we used a novel mitophagy inducer, TJ0113, to investigate the effects and mechanisms of TAA-induced ALF mice. The results showed that TJ0113 could enhance mitophagy through Parkin/PINK1 and ATG5 pathways, which in turn attenuated mitochondrial damage, hepatocyte apoptosis, nuclear factor (NF)-κB/NLRP3 signaling activation and inflammatory responses after TAA. Metabolomics results showed that TJ0113 mainly regulated lipid metabolism, amino acid metabolism and nucleotide metabolism in the livers of ALF mice. RNA sequencing (RNA-seq) analysis yielded that TJ0113 was involved in the development of ALF by regulating the P13K/AKT signaling pathway. The key highlight of this work is the use of an aberration-free line-scanning confocal Raman imager (AFLSCRI) to study the molecular changes in blood, liver tissue, gastrocnemius muscle, and mitochondrial extracts in ALF mice after TJ0113 treatment. Compared to the measurement with conventional assays, Raman microspectroscopy (micro-Raman) offers the benefits of being rapid, non-invasive, label-free and real-time. Our results found good agreement between Raman signals and histopathologic findings. The system has good performance with a spatial resolution of 2 μm, a spectral resolution of 4 cm^−1^ and a fast detection speed improved by 2 orders. Innovations in this test contribute to clinical diagnosis of disease, personalized treatment, effective intraoperative guidance and accurate prognosis. The data may help in the development of a non-invasive clinical device for mitochondrial damage using bedside micro-Raman.

## Highlights

• In vivo Raman spectroscopy enables rapid and noninvasive detection of mitochondrial redox status in TAA-induced ALF mouse models, offering advantages over conventional invasive detection methods.
• The changes in mitochondrial dysfunction during ALF in mice can be detected in real time using Raman microscopy and provide a biomarker that can be used as a specific target for adjuvant therapy.
• TJ0113, a novel mitophagy inducer, attenuates the progression of ALF in mice. TJ0113 induces mitophagy to inhibit the activation of NLRP3 inflammatory vesicles ameliorates apoptosis and inflammatory response in ALF in mice.
• TJ0113 ameliorates ALF progression by modulating lipid metabolism and P13K/AKT pathway in the liver of TAA-induced ALF mice.

## Graphical abstract

**Study on the workflow and mechanism of mitophagy induced by TJ0113 based on mirco-Raman. Workflow diagram**: blood, liver tissue, gastrocnemius muscle, and mitochondrial extracts from liver tissue were obtained from different groups of mice for micro-Raman measurement. Based on micro-Raman to collect many Raman signals, the therapeutic effect and mitophagy alteration of TJ0113 could be clarified. **Schematic diagram of the mechanism:** sustained TAA stimulation leads to extensive mitochondrial damage and sustained activation of the NF-kB/NLRP3 pathway in damaged mitochondria. TJ0113 induces mitophagy by acting on Mcl-1 via the PINK1/parkin pathway, which then binds to LC3A. TJ0113 inhibits inflammatory responses and NLRP3 inflammasome activation, thereby ameliorating the progression of ALF. In addition, RNA sequencing analysis showed that TJ0113 regulated the PI3K/AKT signaling pathway to improve the progression of ALF.

**Figure:**
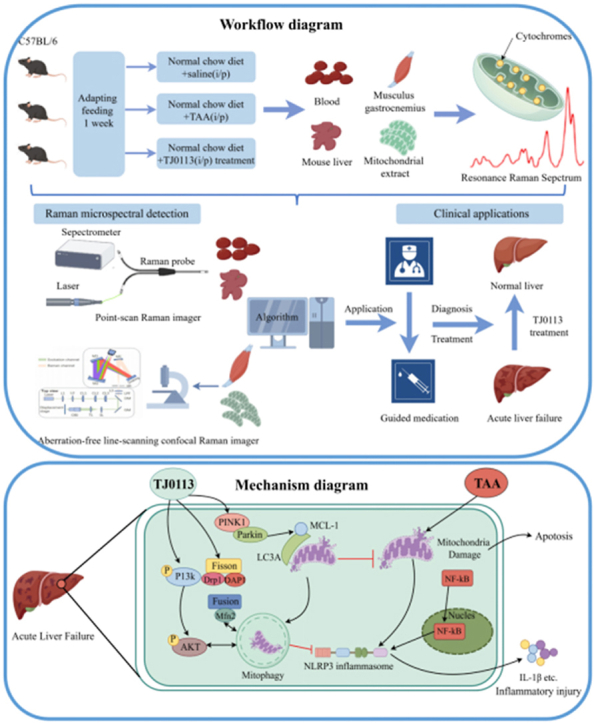

## 1 Introduction

The liver is a major organ for drug metabolism and excretion. Drug-induced liver injury has become a major cause of acute liver failure (ALF) and transplantation [1]. ALF is a medical emergency, and fulminant functional hepatic insufficiency leads to rapid clinical deterioration and high mortality [2,3]. However, liver transplantation is limited by the rapid progression of ALF, the high cost of surgery, and the shortage of donor livers. In addition to thioacetamide (TAA) [4], established mouse models utilizing lipopolysaccharide (LPS)/d-galactosamine (D-GaIN), acetaminophen (APAP) [5], and carbon tetrachloride (CCL4) are also commonly used in modeling ALF [6]. Regardless of the etiology, severe hepatocellular mitochondrial dysfunction and a persistent hepatic inflammatory response are the main features of ALF [[7], [8], [9]]. Therefore, enhancing mitophagy and modulating hepatic inflammation is a promising strategy for the prevention and treatment of ALF [3,10].

Mitochondria are central players in cellular energy metabolism. They participate in necrotic cell death and programmed apoptosis, and are crucial for cell metabolism and survival [11]. Recent studies have found that mitochondria are key drivers of cell death-associated inflammation, and that damaged mitochondria can activate NLRP3 inflammatory vesicles and initiate pro-apoptotic mechanisms, leading to apoptosis [12,13]. Mitophagy prevents the generation of mitochondrial reactive oxygen species (ROS) and the initiation of the mitochondrial apoptotic pathway by selectively removing damaged and excess mitochondria [14,15]. In ALF caused by heat stroke, Huang et al. found that overexpression of P53 and silencing of Parkin could inhibit mitophagy to aggravate ALF [16]. Mitochondria play a central role in regulating cell death and ALF induced by various drugs. Timely clearance of damaged mitochondria is critical for the progression of ALF, whether the ALF is induced by acetaminophen, alcohol or other etiologic causes [8,17]. However, there is a lack of effective mitophagy inducers, and it is unclear whether pharmacologic activation of mitophagy can inhibit ALF.

UMI-77 was originally recognized as a Mcl-1 inhibitor that inhibits cancer cell growth and induces apoptosis [18]. Mcl-1 is a recently discovered mitophagy receptor that interacts with LC3A on the mitochondrial surface to initiate mitophagy [19]. It was demonstrated in Alzheimer's disease mice, sepsis, lupus nephritis, and renal fibrosis mice that UMI-77 slowed down disease progression by activating mitophagy. UMI-77 showed the potential to induce mitophagy, suggesting that Mcl-1 may mediate mitophagy activation [[20], [21], [22], [23]]. Most known mitophagy inducers, such as CCCP, were found in previous studies to force cells to undergo mitophagy mainly by damaging mitochondria. Cen et al. found that UMI-77 did not damage mitochondria, instead, UMI-77 could cause selective degradation of damaged mitochondria [20]. TJ0113 is a derivative of UMI-77. TJ0113 has better drug-forming properties and stronger selective autophagy induction than UMI-77 [24].

Current methods of assaying mitochondrial function require isolation and purification of mitochondria or the use of fluorescent probes, which may interfere with normal mitochondrial function [6,25,26]. Raman microspectroscopy (micro-Raman) is a label-free, non-invasive detection technique with sharp, well-defined spectral peaks that can identify molecules by their unique vibrational fingerprints, making it more suitable for quantitative studies [27,28]. Micro-Raman is an attractive alternative to *in vivo* spectroscopic studies, offering high lateral and spectral resolution, as well as sensitivity and specificity more than 90 % [29,30]. Compared to traditional assays, micro-Raman is not only noninvasive but also allows for real-time detection of mitochondrial changes. Many recent studies have found that micro-Raman (often combined with surface-enhanced chips to improve the Raman signals) can be used to assay mitochondrial function in isolated cells, tissues and blood [31,32]. Therefore, a high signal-to-noise ratio (SNR) micro-Raman (without invasive surface-enhanced chips) measurement of mitophagy is a promising approach to quantify the alteration of mitophagy during drug therapy for ALF.

In this study, we used two Raman imaging systems, a conventional point-scan Raman imaging system and an aberration-free line-scanning confocal Raman imager (AFLSCRI) developed by us. The AFLSCRI system has good performance with a spatial resolution of 2 μm, a spectral resolution of 4 cm^−1^ and a detection speed which is 2 orders of magnitude faster than the conventional system. AFLSCRI was developed in our previous study to identify various types and sizes of microplastics [33]. Here, we used our AFLSCRI of high SNR (without the assistance of any invasive surface-enhanced chip) to assess the mitophagy status in the blood and tissues of ALF mice and further investigated the ameliorative effects of TJ0113 treatment on mitochondrial function, organ damage, hepatocyte apoptosis and inflammatory response.

## 2 Materials and methods

### 2.1 Raman microspectroscopy (micro-Raman)

#### 2.1.1 Blood sample testing system setup

Under 532 nm excitation, cytochrome (cyt) c and cyt *b* can be selectively enhanced through resonance enhancement effects, and are clearly visible through Raman spectroscopy. Therefore, we chose 532 nm as the laser source wavelength for the blood sample detection system [33].

To quickly obtain preliminary data and verify the effectiveness of the experimental scheme, we built a blood sample detection system using a traditional point-wise Raman detection system. The system includes a single-mode fiber-coupled 532 nm laser (FC-D-532-400mW, Changchun) and a fiber-optic Raman spectrometer (QE Pro-Raman, Ocean Insight), connected to the excitation and collection fibers of a Raman fiber probe (RPB-532, Ocean China). The laser output power and spectrometer integration time were adjusted to optimize the signal-to-noise ratio. The probe tip was placed close to or in contact with the sample surface, maintaining a certain working distance to avoid damaging the probe or the sample. The laser and spectrometer were then activated to collect, observe, and record Raman scattering signals. If measurements were taken at different samples, the above steps were repeated until all measurements were completed. Ten Raman spectra were measured for each sample under 532 nm excitation, averaged over a collection time of 15 s, with a laser power of 25 mW.

#### 2.1.2 System setup for *in vivo* testing gastrocnemius muscle

In this experiment, we used the AFLSCRI from our previous paper, based on the experimental results, under the current configuration and sample conditions, the spectral resolution of the AFLSCRI system is 4 cm-1, and after calibration, the system achieves a spatial resolution of 2 μm [32]. Moreover, the high-sensitivity confocal architecture inherently provides a tomographic effect. By applying displacement along the depth dimension, signals from different depths can be captured slice by slice, resulting in a valid imaging depth up to 50–100 μm [70,71]. This device has been optimized to meet the high sensitivity and accuracy requirements for *in vivo* gastrocnemius muscle detection. This not only validates the device's reliability and applicability but also ensures the comparability of experimental data.

The system comprises three core components: the laser line generation unit, a custom-designed aberration-corrected spectrometer, and a confocal scanning microscope. The spatial resolution can reach 2 μm, and the spectral resolution can reach 0.2 nm. Shorter-wavelength excitation light can produce higher Raman signals but is more likely to induce strong fluorescence signals, which can damage biological samples. Longer wavelengths are better to avoid fluorescence phenomena and biological tissue damage, but the Raman signal is weaker, which might lead to ineffective signal detection. Considering the trade-offs between fluorescence, Raman signal-to-noise ratio, and biological damage, we ultimately chose 671 nm instead of 532 nm (used in blood sample detection) as the excitation wavelength.

Raman spectra of the gastrocnemius muscle of each mouse were measured along five lines under 671 nm excitation, with an exposure time of 15 s. The overall power of the line laser is 150 mW.

#### 2.1.3 Raman spectral of blood samples

Real-time blood samples were obtained from mice (4 mice per group) via the tail vein, with Raman spectra measurements taken at a minimum of 10 random points per sample. The Raman signals of blood samples were recorded at different time points post-modeling, and the mean spectrum for each group was presented. At the end of the experiment, the liver tissues of these mice were collected, and their Raman spectra were recorded.

Raman signal detection was conducted at 8 h, 18 h, 24 h, 27 h, and 30 h post-modeling, with groups divided as follows: control group, TAA group, TAA + TJ0113 (1 mg/kg) group, TAA + TJ0113 (10 mg/kg) group, and TJ0113 (10 mg/kg) group.

#### 2.1.4 Raman spectral of gastrocnemius muscle

After anesthetizing the mice with sodium pentobarbital, they were fixed on a splint with elastic glue. The hair in the gastrocnemius muscle area was shaved off, and the skin surface was removed with tweezers. The objective height was adjusted to properly focus the laser spot on the surface of the mouse gastrocnemius muscle. Subsequently, line imaging was performed using an AFLSCRI. The mice were divided into three groups: the TAA model group, the TAA + TJ0113 (1 mg/kg) group, and the control group, with 3 mice in each group. Raman detection of the gastrocnemius muscle was performed at 12 h, 16 h, 20 h, 22 h, and 24 h post-modeling, with measurements taken from three fixed positions on the surface of the mouse gastrocnemius muscle each time. Since the control group mice were not modeled, they were only measured once after the other mice to indicate the normal level of gastrocnemius muscle Raman signals.

#### 2.1.5 Assessment of the therapeutic effects of TJ0113 on mice based on gastrocnemius muscle Raman detection

To investigate whether the timing of TJ0113 administration has a preventive or therapeutic effect on the symptoms of liver failure in mice, five groups were set up: administration 1 h before modeling, administration 4 h after modeling, administration 12 h after modeling, a modeling group, and a control group. Raman detection of the gastrocnemius muscle was conducted for all groups. The first four groups of mice underwent gastrocnemius muscle Raman detection at 14 h, 17 h, 20 h, 22 h, 24 h, 26 h, and 28 h post-modeling. For the control group, one session of gastrocnemius muscle Raman detection was performed after all other mice were tested.

#### 2.1.6 Extraction of mitochondria from liver tissue for Raman detection

Mitochondria were isolated from liver tissue using the MinuteTM Mammalian Cell/Tissue Mitochondrial Isolation Kit (Cat# MP-007, Invent, Beijing, China). The isolation of mitochondria was performed according to the instructions of the kit, and the mitochondria obtained from the isolation were observed under a Raman microscope and the Raman signal was recorded.

### 2.2 Animal experimentation

#### 2.2.1 Experiment animals and drug treatment

8–10 weeks old male C57BL/6 mice, weighing 23–25 g, were purchased from Hangzhou Hangsi Biotechnology Co., Ltd (Hangzhou, China). All mice were housed in a specific pathogen-free environment with a light cycle of 12 h/a dark cycle of 12 h, and fed ad libitum with standard normal chow and water. Animal experiments were approved by the Animal Experimentation Ethics Committee of Taizhou Hospital, Zhejiang Province (Approval No. tzy-2023216). The 3Rs guidelines for animal welfare were followed for *in vivo* studies.

As a common hepatotoxin, TAA has been extensively used animal models of ALF [1,29]. After 1 week of acclimatization feeding, the mice were divided into 7 groups (12 mice per group): control group (intraperitoneal injection of 0.1 % NaHCO3); TAA group (intraperitoneal injection 800 mg/kg TAA); TAA + TJ0113 groups (TAA + gavage injection (5 mg/kg or 10 mg/kg) TJ0113 or intraperitoneal injection (0.5 mg/kg or 1 mg/kg) TJ0113); TJ0113 group (gavage injection 10 mg/kg TJ0113). Pre-treatment of TJ0113 was given 1hr before TAA modeling. TJ0113 can be dissolved in 0.1 % NaHCO3. 6 mice were euthanized 24 h after TAA modeling, and blood and liver tissues were removed for further analysis. Tissues were fixed with 4 % paraformaldehyde or glutaraldehyde and subjected to hematoxylin-eosin (H&E) staining, immunofluorescence (IF), immunohistochemistry (IHC) or immediate RS assay. The remaining mice were subjected to the micro-Raman assay.

#### 2.2.2 Survival experiment

To investigate the effect of TJ0113 on the survival of TAA-treated mice, mice were divided into control, TAA, TAA + TJ0113 (0.5 mg/kg), TAA + TJ0113 (1 mg/kg), TAA + TJ0113 (5 mg/kg), TAA + TJ0113 (10 mg/kg), TJ0113 (10 mg/kg), 10 per group. After the intervention of TAA and TJ0113 injection, the mortality rate of animals in each group was observed every 4 h and the number of surviving animals was recorded for consecutive 36 h.

#### 2.2.3 Biochemical analyses

Blood samples were collected from mice after anesthesia, placed in non-anticoagulated tubes, and the serum was separated by centrifugation at 4000 rpm for 10 min at 4 °C. Serum alanine aminotransferase (ALT, Cat# C009-2-1) and aspartate aminotransferase (AST, Cat# C00-2-1) were measured using biochemical assay kits to assess liver injury (Nanjing Jiancheng Bioengineering Institute, Nanjing, China). Serum interleukin-1β (IL-1β, Cat# E-MSEL-M0003), tumor necrosis factor-α (TNF-α, Cat# E-MSEL-M0002), and interleukin-6 (IL-6, Cat# E-MSEL-M0001) levels were determined using enzyme-linked immunosorbent assay (ELISA) kits according to the manufacturer's protocols (Elabscience, Wuhan, China).

#### 2.2.4 Histopathological analysis and immunohistochemistry

Fresh liver tissues were fixed in 4 % neutral buffered formalin for 24 h. Liver specimens were sectioned to 5 μm thickness. Sections were stained with hematoxylin and eosin according to standard histologic methods. Liver pathologic changes were observed and evaluated under light microscopy. As described in previous studies, a liver histology score was used to determine the degree of liver injury, which was divided into an inflammation score and a necrosis score [4,5].

For immunohistochemistry, paraffin-embedded liver tissue sections were subjected to the following: degumming, rehydration, and antigen recovery by boiling sections in sodium citrate buffer (10 mM sodium citrate, 0.05 % Tween 20, pH 6.0) and blocking (1 % bovine serum albumin (BSA), 1 h) at room temperature (RT). Sections were then incubated with the primary antibodies, 8-hydroxyguanosine (8-OHdG, ab62623, Abcam, 1:500) and Autophagy Related 5 (ATG5, ab108327, Abcam, 1:500). The samples were left at 4 °C overnight and then incubated with the appropriate secondary antibody. The prepared DAB chromogenic solution working solution was added dropwise to the tissue sections, and the color was developed for 10 min and then observed microscopically. The stained area was calculated using ImageJ software.

#### 2.2.5 TUNEL (TdT-Mediated dUTP nick end labeling)

Hepatocyte apoptosis assay was performed using the TUNEL kit (Roche, USA). Liver sections were incubated with proteinase K (Roche, USA) for 15 min at 37 °C and then washed with phosphate buffered saline (PBS). Sections were incubated with TUNEL reaction mixture at 37 °C for 1 h. Cell nuclei were stained with DAPI for 5 min (Beyotime, China). TUNEL-positive cells were observed and analyzed by confocal microscopy.

#### 2.2.6 Immunofluorescence staining

Frozen liver tissue sections were fixed with 4 % polyformaldehyde (PFA), permeabilized with 0.3 % Triton X-100 for 20 min, and then blocked with 5 % BSA for 1 h. The primary antibodies used in this study included: MCL1 apoptosis regulator (Mcl-1, Cat# ab32087, Abcam), microtubule associated Protein 1 Light Chain 3 Alpha/Beta (LC3A/B, Cat# 4108, Cell Signaling Technology), mitochondrial outer membrane 20 (TOM20, Cat# 42406, Cell Signaling Technology), NLR family pyrin domain containing 3 (NLRP3, Cat# ab270449, Abcam) and lysosome-associated membrane protein 1 (LAMP1, Cat# 99437, Cell Signaling Technology). The samples were then incubated in 1 % BSA-coated specific primary antibodies at 4 °C overnight. The next day, the primary antibody was washed away and incubated with the corresponding secondary antibody (Servicebio, Wuhai, China) for 1 h at room temperature under light-protected conditions. DAPI was used for nucleation. Images were captured on a confocal microscope (Nikon A1).

#### 2.2.7 Western blot

Liver tissues were lysed with radioimmunoprecipitation assay buffer (RIPA, Cat# P0013B Beyotime Biotechnology) containing a mixture of protease and phosphatase inhibitor (PMSF, Cat# P1045, Beyotime Biotechnology) and total protein was extracted. Protein concentration was determined by Bicinchoninic Acid Assay (BCA) protein assay kit (Cat# P0011, Beyotime). 15–20 μg of protein was separated by electrophoresis on sodium dodecyl sulfate-polyacrylamide gel (SDS-PAGE) and transferred to a polyvinylidene difluoride (PVDF) membrane (Millipore, USA). The membrane was blocked with 5 % skim milk and incubated with primary antibody overnight at 4 °C, followed by incubation with horseradish peroxidase (HRP) -labeled secondary antibodies for 1 h at room temperature. Wash three times with TBST for 5 min each time. Cutting the membrane horizontally according to the position of the target protein, protein-antibody complexes were detected on an ImageQuant LAS-500 imaging system (GE Health Care, USA) using enhanced chemiluminescence (ECL) reagents (Millipore, USA). Finally, ImageJ software (NIH, USA) was used to quantify and analyze the bands. The primary antibodies used in this study included: β-actin (Cat# AY0573, 1:1000), PINK1(Cat# BY0130, 1:1000), Parkin (Cat# CY6641, 1:1000), P65(Cat# CY5034, 1:1000), OPA1(Cat# CY7035, 1:1000), Mitofusin 2 (Mfn2, Cat# CY6638, 1:1000), Bax (Cat# CY5059, 1:1000), caspase-3(Cat# CY5384, 1:1000) were purchased from Abways (Shanghai, China). Pro-Caspase-3(Cat# ab32499, 1:1000), Bcl-2(Cat# ab182858, 1:1000), Drp1(Cat# ab184247, 1:1000), ASC(Cat# ab309497, 1:1000), IL-1β (Cat# ab283818, 1:1000), Mcl-1(Cat# ab32087, 1:1000) were purchased from Abcam (Cambridge, the UK). P13K(Cat# 4292, 1:1000), p-P13K(Cat# 4228, 1:1000), AKT (Cat# 9272, 1:1000), *p*-AKT (Cat# 4060, 1:1000), NLRP3(Cat# 15101, 1:1000), LC3A/B (Cat# 4108, 1:1000) were purchased from Cell Signaling Technology (Boston, USA).

#### 2.2.8 Transmission electron microscopy (TEM)

1 mm^3^ of fresh liver tissue was taken and immediately fixed with 2.5 % glutaraldehyde. Then, 0.1 M phosphate buffer PB (PH7.4) formulated with 1 % osmium acid was fixed at room temperature away from light for 2 h, dehydrated with ethanol and permeabilized with acetone and embedded overnight at 37 °C. The samples were polymerized for 48 h at 60 °C. The sample resin blocks were stained with toluidine blue and localized under a light microscope, then cut into ultrathin sections, stained with uranyl acetate and lead citrate, and subsequently examined under a transmission electron microscope (HITACHI HT7700 80kv, Japan).

#### 2.2.9 RT-qPCR

PrimeScrip™ RT kit from Takara, Japan and polymerase chain reaction equipment from Bio-Rad, USA were used. An ABI7500 real-time PCR instrument (Applied, USA) and TB Green®PreMix Ex Taq™II (Takara, Japan) were used to amplify cDNA. The cycling threshold (Ct) was calculated and normalized to the level of housekeeping genes (18s). The expression of each group of target genes were detected by the 2^−ΔΔCt^ method. The primer sequences were shown in Table 1.

**Table 1:** Primer sequence.

Species	Primer name (Forward/Reverse)	Primer sequence (5′-3′)
Mus	Mcl-1-F	ATGGCTGAGTGGGACATCGC
Mus	Mcl-1-R	TGCTCCTCAGCTTGGGAGGT
Mus	LC3A-F	TGGCTGTTGTTGAGTGGTCT
Mus	LC3A-R	TCACCTCGTCCGATAGTTCG
Mus	GAPDH-F	ACAACTTTGGTATCGTGGAAGG
Mus	GAPDH-R	GCCATCACGCCACAGTTTCC

#### 2.2.10 RNA sequencing and analysis

Total RNA was extracted from different groups of liver tissues to isolate mRNA, and fragmented RNA was obtained by treatment with lysis buffer, and double-stranded cDNA was synthesized by using random hexamer primers, buffer, dNTPs, and DNA polymerase I. Purified double-stranded cDNA was ligated at the end with an “A” tail. Finally, the enriched cDNA library was generated by PCR amplification. Preliminary quantification was performed using Qubit 2.0 and the insert size of the library was evaluated on an Agilent Bioanalyzer 2100 system. Sequencing was also performed using the Illumina platform. Differential genes were screened by |log2FC| ≥ 1 and P-value≤0.05. Gene Ontology (GO) enrichment analysis including Molecular Function (MF), Biological Process (BP) and Cellular Component (CC). Kyoto Encyclopedia of Genes and Genomes (KEGG) pathway enrichment was performed using R Language.

#### 2.2.11 Liver metabolomics analysis

The separation was performed on an ACQUITY UPLC® HSS T3 (2.1 × 100 mm, 1.8 μm) (Waters, Milford, MA, USA) column. The injection volume was 16 μL, the column temperature was 40 °C, and the flow rate was 0.3 mL/min. The mobile phase consisted of two parts: phase A (ultrapure water plus 0.1 % formic acid) and phase B (acetonitrile plus 0.1 % formic acid). A gradient was established during the elution process. Each sample was detected by electrospray ionization (ESI) in positive (+) and negative (−) mode. Samples were separated by UPLC and analyzed by mass spectrometry using a QE Plus mass spectrometer (Thermo Scientific). Raw data were analyzed by MSDIAL software for peak alignment, retention time correction and peak area extraction. The structural identification of metabolites was performed by precise mass number matching (mass tolerance <10 ppm) and secondary spectral matching (mass tolerance <0.01 Da), and the public databases, such as HMDB, MassBank, GNPS, and the self-constructed metabolite database (BP-DB), were searched. The data were pre-processed by Unit variance scaling (UV) for subsequent differential metabolite and biofunctional enrichment analysis.

#### 2.2.12 Statistical analysis

All data are expressed as mean ± standard deviation (SD). Data were analyzed by ANOVA using the Prism 9 software package (GraphPad software, La Jolla, CA). Differences were compared using one-way ANOVA followed by Tukey post hoc tests. Kaplan-Meier survival analyses were performed using Log-rank tests. P < 0.05 was considered statistically significant. All statistics were obtained from at least three independent biological replicates.

## 3 Results

### 3.1 TJ0113 attenuates TAA-induced mitochondrial damage in ALF mice

To validate the effectiveness of micro-Raman in detecting mitochondrial damage in ALF mice, we first used a 532 nm laser for continuous detection of mouse blood. At this wavelength, cytochrome *b* (cyt *b*) and cytochrome *c* (cyt *c*) are selectively enhanced, with their corresponding Raman characteristic peaks at 750 cm^−1^, 1128 cm^−1^, and 1585 cm^−1^, representing the symmetric vibration of porphyrin, the C_b_-CH_3_ side radical, and the methyl bridge (C_a_C_m_, C_a_C_m_H bond) vibration, respectively.

**Fig. 1:**
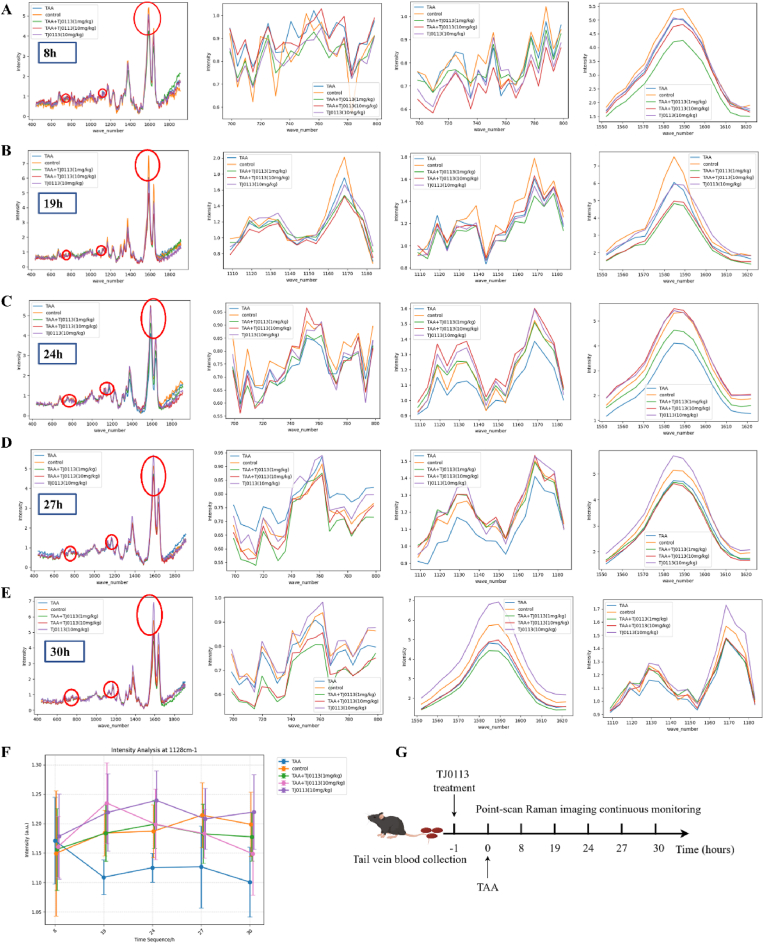
Raman signal variations in the blood of different mouse groups **over time.** (A–E) Show the results of Raman detection of the tail vein blood of mice at 8 h, 18 h, 24 h, 27 h, and 30 h, respectively. Each set of images includes four graphs covering the ranges from 400 cm^−1^ to 1800 cm^−1^, 700 cm^−1^ to 800 cm^−1^, 1110 cm^−1^ to 1180 cm^−1^, and 1550 cm^−1^ to 1620 cm^−1^. (F) Depicts the normalized intensity of the peak at 1128 cm^−1^, with 1008 cm^−1^ as the reference, showing the time-dependent changes in this characteristic peak for the five groups of mice. (G) micro-Raman continuous monitoring timeline.

Given the complexity of blood Raman signals, we selected the phenylalanine peak at 1008 cm^−1^ as a normalization standard due to its relative stability across different samples, representing the stable conformational changes of proteins. To investigate whether TJ0113 improves mitochondrial damage in liver failure mice, we administered TJ0113 1 h before TAA modeling and divided the mice into five groups: control group, TAA group, TAA + TJ0113 (1 mg/kg) group, TAA + TJ0113 (10 mg/kg) group, and TJ0113 (10 mg/kg) group. 1 mg/kg TJ0113 was administered by intraperitoneal injection to mice. 10 mg/kg was administered by gavage. Blood samples were collected from the tail vein at 8 h, 18 h, 24 h, 27 h, and 30 h post-modeling for Raman spectroscopy (Fig. 1G).

The experimental results showed that at different time points, the baseline Raman signals of mice in each experimental group were highly consistent. This indicates that the mice had good uniformity in their baseline state, and the grouping was reliable (Fig. 1A–E). Considering the weaker Raman signal at 750 cm^−1^ and potential interference at 1585 cm^−1^, we ultimately used the normalized 1128 cm^−1^ peak as the Raman detection indicator for mitochondrial damage in mouse blood. The changes over time are shown in Fig. 1F. The control group maintained relative stability, while the TAA group showed a rapid decline in signal post-modeling, indicating TAA-induced mitochondrial redox dysfunction and decreased cytochrome content. In contrast, the three groups treated with TJ0113 exhibited a trend of initial increase followed by a decrease in Raman signal, with a peak at 24 h post-modeling higher than the control group, followed by a decline to near-control levels at 30 h. These results suggest that TJ0113 induces beneficial mitophagy effects in ALF mice, with time-dependent efficacy.

**Fig. 2:**
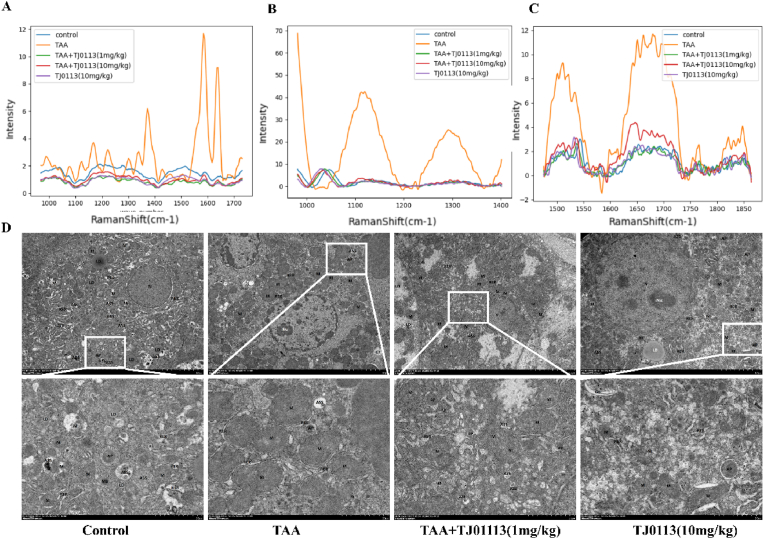
Raman signals from liver tissue and mitochondrial extracts as well as images of liver t**issue TEM.** (A) Raman signals of liver tissue samples from five different mouse groups. (B) Raman signal (1000-1400 cm^−1^) of mitochondrial extracts from five different mouse groups. (C)Raman signal (1450-1870 cm^−1^) of mitochondrial extracts from five different mouse groups. (D) Representative TEM images of TAA-induced mitochondrial morphology in hepatocytes of ALF mice. 3000× magnification (top) and 8000× (bottom) magnification; Scale bar: 5 μm and 2 μm. Nucleus (N); Nucleolus (Nu); Mitochondria (M); Rough endoplasmic reticulum (RER); Lipid droplets (LD); Autophagic vesicles (AP); Autophagic lysosomes (ASS); Microsomes (MB); Collagen fibers (CF); Golgi apparatus (GO).

After the blood sample detection experiment, liver tissue sections from the above mice were obtained and their Raman spectra were recorded, as shown in Fig. 2A. There was a significant difference in the TAA group compared with the other four groups, and a distinct cytochrome characteristic peak was detected in the liver tissue. The mitochondrial damage in liver tissues of TAA group was quite severe compared with other groups. In contrast, the intensity of the characteristic peaks in the three groups of mice treated with TJ0113 was close to that of the control group. We isolated and extracted mitochondria from liver tissue and obtained similar results using micro-Raman detection (Fig. 2B–C). Among the liver tissues and liver tissue mitochondrial extracts, mitochondrial damage in liver tissues of mice treated with 10 mg/kg TJ0113 by gavage was slightly more severe compared with 1 mg/kg TJ0113 by intraperitoneal injection. We used a transmission electron microscope (TEM) to directly observe the mitochondrial morphology. We found that the mitochondrial morphology of hepatocytes was disrupted in the liver tissues of ALF mice, as evidenced by mitochondrial swelling, vacuole formation and cristae loss as well as the presence of partially visible mitochondrial autophagic lysosomes. Mitochondrial morphology was partially restored after TJ0113 treatment, as evidenced by mild swelling of mitochondria, minor cristae breaks and increased mitochondrial autophagic lysosomes (Fig. 2D).

### 3.2 Continuous monitoring of mouse gastrocnemius muscle by AFLSCRI

**Fig. 3:**
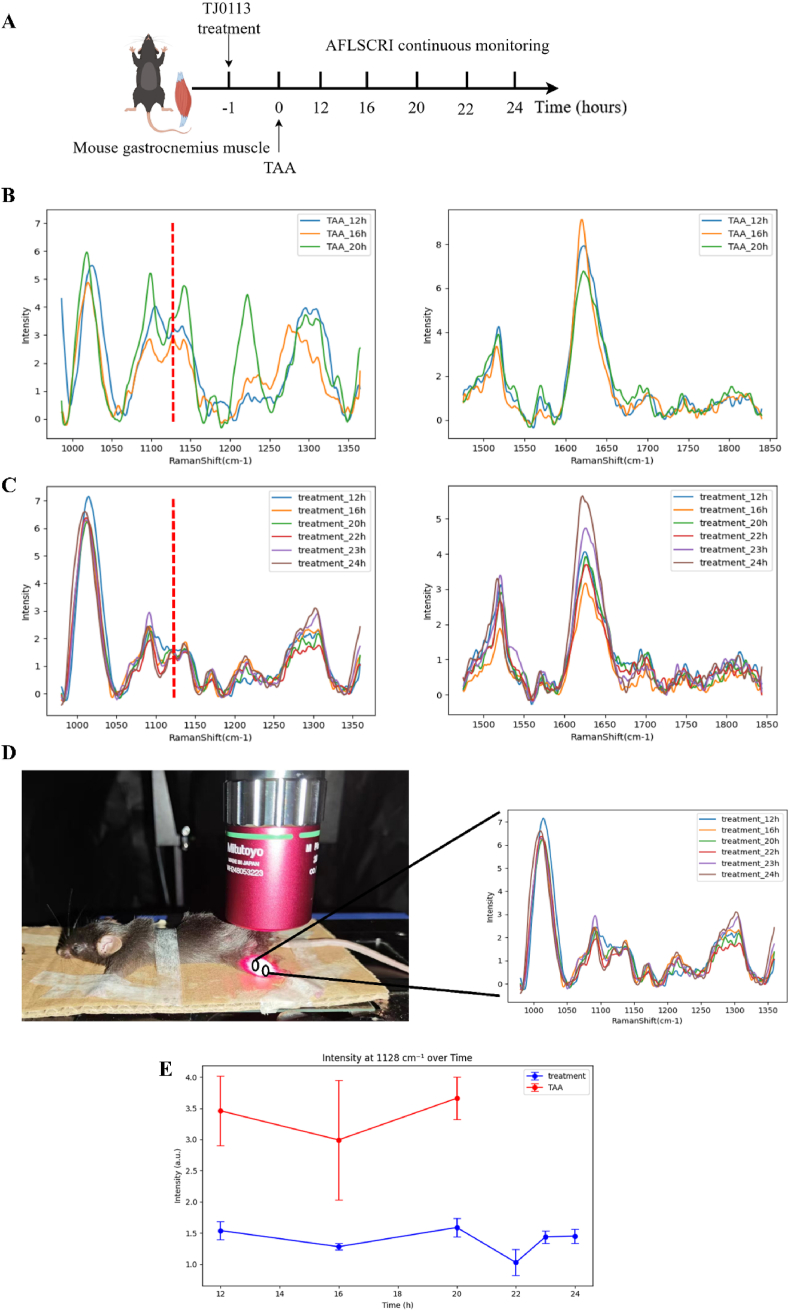
Temporal variations in Raman signals at gastrocnemius muscles of two group**s of mice.** (A) Timeline. (B) Raman signals of the gastrocnemius muscle in TAA group mice. (C) Raman signals of the gastrocnemius muscle in TJ0113-treated group mice. (D) One mouse anesthetized and fixed under our line scanning confocal Raman imager for Raman imaging. (E) The time variation of the normalized Raman signal of the gastrocnemius muscle at 1128 cm^−1^ for two groups of mice.

To achieve non-invasive detection of the gastrocnemius muscle in mice, we used a 671 nm laser source to minimize fluorescence interference while ensuring strong enough Raman signal. The experimental mice were divided into the TAA group and the TJ0113 treatment group. The treatment group received TJ0113 1 h before receiving TAA (Fig. 3A). The Raman signals at the gastrocnemius muscle in mice are shown in Fig. 3B. The TAA group exhibited significant fluctuations in the Raman characteristic signal at 1128 cm^−1^, maintaining a relatively high level compared to the intervention group, consistent with the results obtained from Raman detection of liver tissues.

In this set of experiments, the TAA group's liver failure symptoms rapidly worsened around 19 h into the experiment. After completing the test at 20 h, all mice in the TAA group died within approximately half an hour. Observations continued for the drug intervention group, with further Raman detection of the gastrocnemius muscle at 22 h, 23 h, and 24 h. Fig. 3D shows a photo of a mouse anesthetized and fixed under our confocal Raman imager. Fig. 3E shows the temporal variation of Raman signal of gastrocnemius muscle at 1128 cm^−1^.

### 3.3 Investigation of the therapeutic effects of TJ0113 on mitochondrial damage in TAA-induced ALF mice

**Fig. 4:**
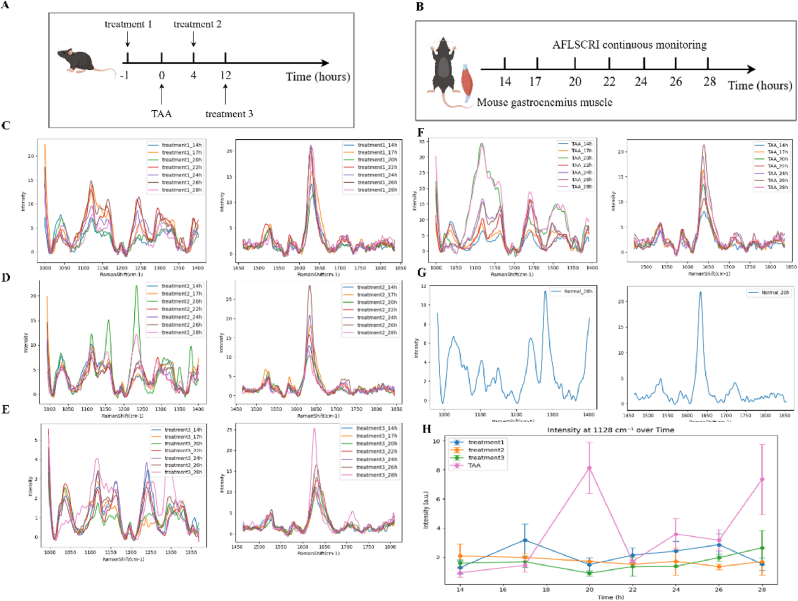
Assessment of the therapeutic effects of TJ0113 on mice based on Raman detection at gastrocnemi**us muscle.** (A) Schematic diagram of *in vivo* experiment design.(B) Raman monitoring flow chart. (C) Raman detection results of the gastrocnemius muscle in mice administered TJ0113 1 h before TAA induction (treatment1). (D) Raman detection results of the gastrocnemius muscle in mice administered TJ0113 4 h after TAA induction (treatment2). (E) Raman detection results of the gastrocnemius muscle in mice administered TJ0113 12 h after TAA induction (treatment3). (F) Raman detection results of the gastrocnemius muscle in TAA group mice. (G) Raman detection results of the gastrocnemius muscle in normal control group mice. (H) The time variation of the normalized Raman signal of the gastrocnemius muscle at 1128 cm^−1^ for four groups of mice.

Based on different intervention times of TJ0113, we divided the mice into 5 groups as shown in Fig. 4A–H to investigate whether TJ0113 has a therapeutic effect on TAA induced ALF symptoms in mice. Using the signal of 1128 cm^−1^ as a characteristic feature, the signal intensity of the three groups intervened with TJ0113 is weaker than that of the TAA group and higher than that of the control group, which once again verifies the effectiveness of TJ0113. Comparing the three groups treated with TJ0113 at different times, it was found that on this characteristic peak, the Raman signal intensity of the gastrocnemius muscle in the treatment three group of mice was relatively lowest, indicating the best intervention effect when testing. This result may provide some guidance for the timing of medication.

### 3.4 TJ0113 enhanced mitophagy and ameliorated mitochondrial damage in ALF mouse hepatocytes

**Fig. 5:**
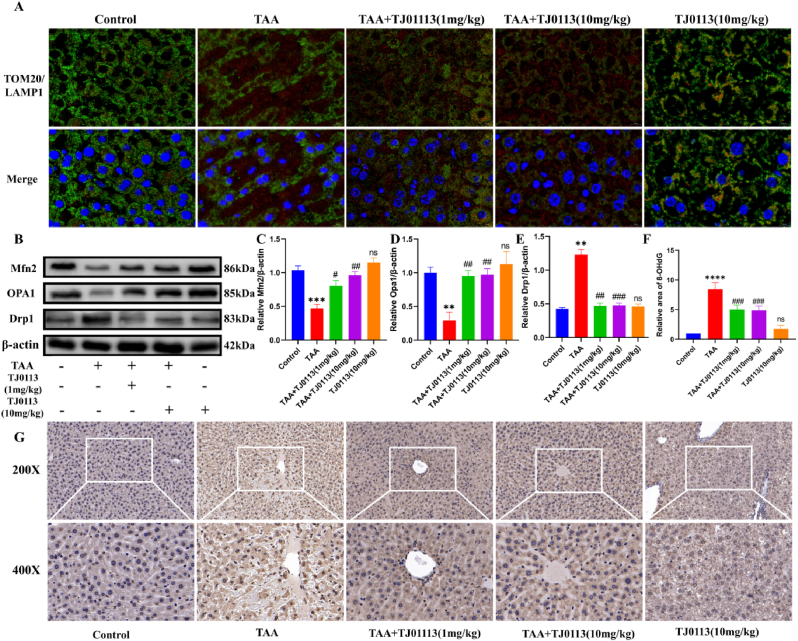
TJ0113 enhanced mitophagy and ameliorated mitochondrial damage in ALF mouse he**patocytes.** (A) Representative images of different groups of ALF mice immune-fluorescently double-labeled with TOM20 (green) and LAMP1 (red), magnified (1000×). (B) Representative immunoblotting images and quantification of mitochondrial dynamics-related proteins Mfn2 (C), OPA1 (D) and Drp1 (E) in liver tissue. (G) Representative IHC images (200X and 400X) and (F) quantification of 8-OHDG positive cells in liver sections. Compared with the control group, ∗∗p < 0.01, ∗∗∗∗p < 0.001. Compared with the TAA group, #p < 0.05, ##p < 0.01, ###p < 0.001; ns, not significant. Data are expressed as mean ± SD.

To evaluate the effect of autophagy inducer TJ0113 on ALF, we used a TAA-induced ALF mouse model. Immunofluorescence staining of TOM20 and LAMP1 showed that autophagosomes were formed in hepatocytes with ALF. In the TAA group, TOM20 and LAMP1 were seen to be inhibited by mitophagy at the severe site of liver tissue injury. Treatment with TJ0113 significantly increases the mitochondrial autophagic lysosomes (Fig. 5A). Double-membrane vesicles containing mitochondria (mitochondrial autophagosomes) were visible in hepatocytes under TEM microscopy (Fig. 2D). In addition, the balance between mitochondrial fission and fusion is closely coordinated with mitochondrial morphology and function. ALF leads to an upregulation of the fission-related protein Drp1, while the fusion-related markers OPA1 and Mfn2 are reduced, indicating an increase in mitochondrial fragmentation (Fig. 5B–E). TJ0113 partially undoes the changes in the mitochondrial dynamics markers. Furthermore, the IHC of the specific oxidative DNA damage marker (8-OHdG) was significantly enhanced after ALF treatment and significantly reduced after TJ0113 treatment, indicating reduced ROS generation and improved oxidative damage (Fig. 5F–G).

**Fig. 6:**
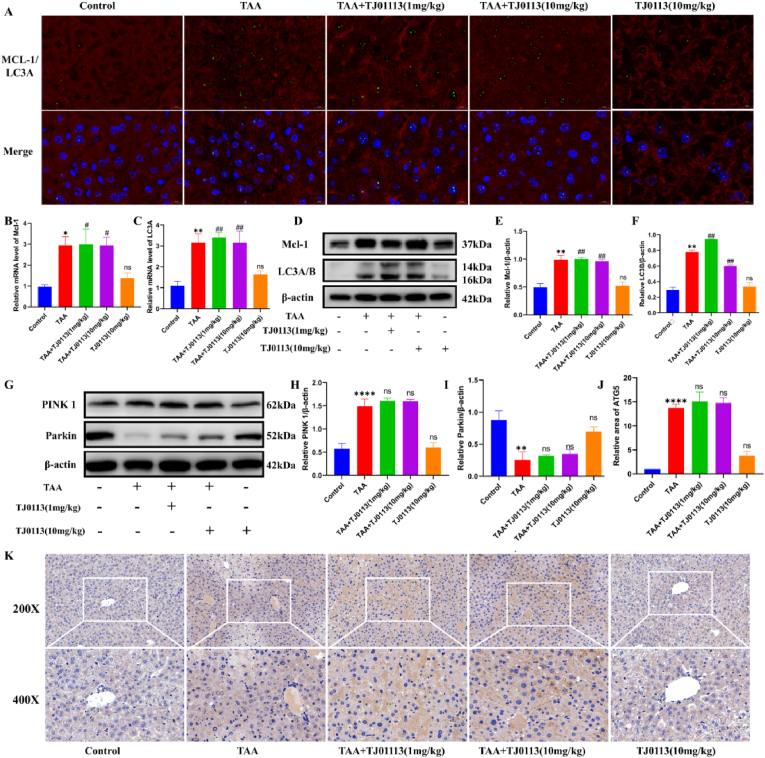
TJ0113 acts on Mcl-1 targets through the PINK1/Parkin and ATG5 pathway and subsequently binds to LC3A to induce **mitophagy.** (A) Representative images of different groups of ALF mice immune-fluorescently double-labeled with Mcl-1 (green) and LC3A (red). The mRNA expression levels of Mcl-1 (B) and LC3A (C) in liver tissues. (D) Representative immunoblotting images and quantification of mitophagy pathway proteins including PINK1(H) and Parkin(I) in liver tissues. (K) Representative immunostaining (200X and 400X) and (J) quantification of ATG5 in liver tissues. Compared with the control group, ∗p < 0.05, ∗∗p < 0.01, ∗∗∗∗p < 0.001. Compared with the TAA group, #p < 0.05, ##p < 0.01, ###p < 0.001; ns, not significant. Data are expressed as mean ± SD.

Mcl-1 is a target of TJ0113 and a mitophagy receptor. Mcl-1 interacts with LC3 to induce mitophagy. Immunofluorescence double labeling was seen to increase Mcl-1 and LC3A binding after TAA and TJ0113 treatment (Fig. 6A). We examined the mRNA expression levels of Mcl-1 and LC3A, which were seen to be significantly elevated in the TAA and TAA + TJ0113 groups (Fig. 6B–C). TAA and TAA + TJ0113 groups resulted in elevated protein expression of Mcl-1 and LC3A/B (Fig. 6D–F). Subsequently, we examined the protein expression levels of the classical and non-classical pathways of mitophagy. The administration of TJ0113 alters the expression of PINK1 and Parkin, suggesting that TJ0113-induced mitophagy is dependent of the PINK1-Parkin pathway (Fig. 6G–I). We conducted IHC experiments on the ATG5 mitophagy bypass pathway and found that ATG5 expression is elevated in ALF, and TJ0113 improves the level of ATG5 (Fig. 6J–K). These results suggest that TJ0113 can activate mitophagy and enhance mitochondrial adaptation through the PINK1/Parkin and ATG5 pathways.

### 3.5 Regulatory effects of TJ0113 on metabolites and P13K/AKT pathway in liver tissue of ALF mice

**Fig. 7:**
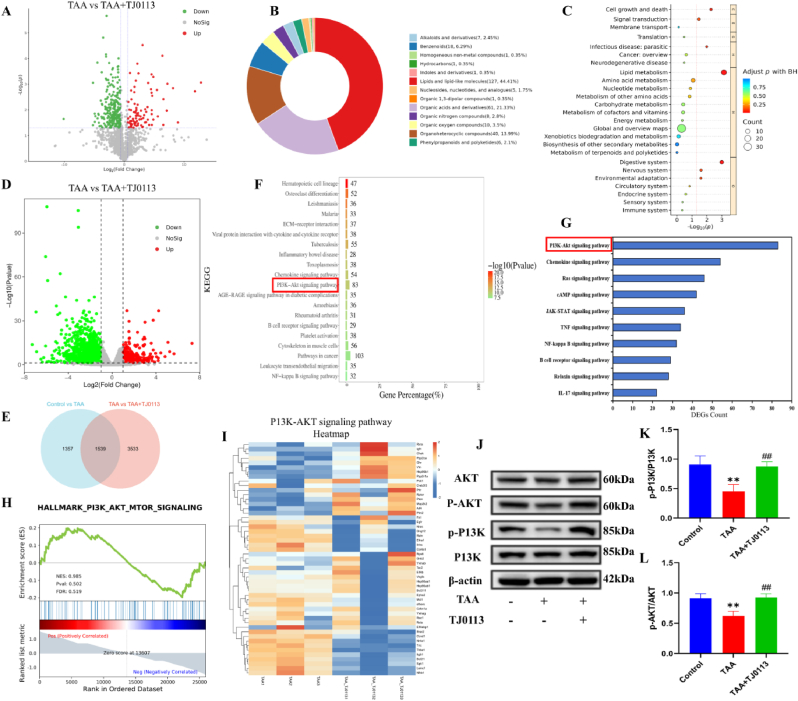
Effects of TJ0113 on hepatic gene and metabolite expression 24 h after TAA injection and TJ0113 can regulate ALF progression through the P13K/AK**T pathway.** (A) Volcano plot of differentially expressed metabolites between TAA and TAA + TJ0113 groups (n = 3/group). (B) Categorical plot of differentially expressed metabolites between TAA and TAA + TJ0113 groups. (C) KEGG analysis of differentially metabolites between TAA group and TAA + TJ0113 group. (D) Volcano plot of differentially expressed genes between TAA and TAA + TJ0113 groups. (E) Venn of the intersection between Control vs TAA and TAA vs TAA + TJ0113 groups. (F) Top20 histogram of KEGG pathway differentially expressed gene enrichment in TAA group vs. TAA + TJ0113 group. (G) Further KEGG enrichment analysis revealed a dominant biological pathway induced by TJ0113 in the livers of TJ0113-treated mice compared to TAA. (H) GSEA analysis of P13K/AKT signaling pathway in TAA and TAA + TJ0113 groups. (I) RNA-seq-based heatmap of the average expression levels of identified genes in the P13K/AKT signaling pathway in TAA group and TAA + TJ0113 group. (J) Representative immunoblotting images and quantification of *p*-AKT/AKT (K) and p-P13K/P13K (L). Compared with the control group, ∗∗p < 0.01. Compared with the TAA group, ##p < 0.01. Data are expressed as mean ± SD.

To systematically demonstrate the role of TJ0113 in ALF, liver tissues from mice in the TAA group and mice in the TAA + TJ0113 group were analyzed by untargeted metabolomics and RNA sequencing (RNA-seq). In metabolomics analysis, we identified differential metabolites based on P ≤ 0.05 and Flog2 (FC) ≥ 1 or ≤ −1. These differentially expressed metabolites were visualized by volcano plots. Compared with the control group, 62 differential metabolites were up-regulated and 161 differential metabolites were down-regulated in the TAA group. Of the 223 differential metabolites, 44.41 % were lipid and lipid-like molecules, 21.33 % were organic acids and their derivatives, and 13.99 % were heterocyclic compounds (Fig. 7B). To discover the most affected metabolic pathways, KEGG pathway analysis was performed using all identified differential metabolites obtained. The TAA + TJ0113 group was enriched in lipid metabolism, amino acid metabolism and nucleotide metabolism compared to the TAA group groups (Fig. 7C). These findings suggest that TJ0113 modulates changes in hepatic metabolites in ALF mice.

Subsequently, we performed RNA sequencing (RNA-seq) analysis of liver tissues from TAA and TAA + TJ0113 groups. We identified the differential genes based on P ≤ 0.05 and Flog2 (FC) ≥ 1 or ≤ −1. A total of 2896 differential genes were brushed out, including 2218 down-regulated genes and 678 up-regulated genes (Fig. 7D). Gene set enrichment analysis showed significant enrichment of biological processes after TJ0113 treatment, including signaling pathways related to immune response and inflammatory response were enriched (Fig. 7F–G). Further Kyoto Encyclopedia of Genes and Genomes pathway analysis showed that the P13K/AKT signaling pathway, which has been shown to play an important regulatory role in ALF, was the most significantly altered pathway after TJ0113 treatment (Fig. 7H–I). The phosphorylation of P13K and AKT was significantly down-regulated by TAA as seen by detecting key molecules of the P13K/AKT pathway, and this alteration was reversed by TJ0113 (Fig. 7J–L). We conclude that TJ0113 is involved in the progression of ALF by regulating the P13K/AKT signaling pathway.

### 3.6 TJ0113 is protective against TAA-induced ALF mice

**Fig. 8:**
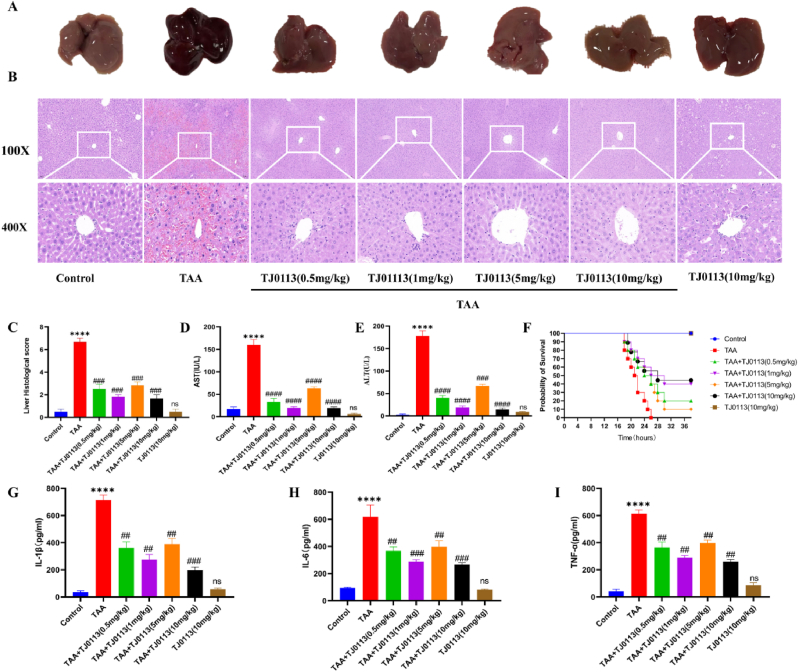
TJ0113 ameliorates liver function and liver injury and improves survival in **ALF mice.** (A–B) Representative images (100X and 400X) of the naked eye view as well as hematoxylin and eosin (H&E) staining of the liver of each group of mice. (C) Hepatic necrosis H&E staining score. At 100× magnification, six ranges were randomly selected and the total score for each section was calculated. Serum levels of AST (D) and ALT (E) in mice in each group. (F) TJ0113 Survival of mice at 36 h in each group at different doses and modes of administration. ELISA for the expression levels of IL-1β (G), IL-6 (H) and TNF-α (I) in mouse serum. Compared with the control group, ∗∗∗∗p < 0.0001. Compared with the TAA group, ##p < 0.01, ###p < 0.001, ####p < 0.0001; ns, not significant. Data are expressed as mean ± SD.

Liver morphology of TAA-induced ALF mice exhibited severe liver injury, whereas there were no significant changes in the control, TJ0113 intervention and TJ0113 control groups (Fig. 8A). H&E staining showed more severe structural damage of the liver tissues, larger necrotic area and increased number of infiltrating inflammatory cells in the model group as compared to the control group. A reduction in the area of hepatic necrosis and injury were seen in the different doses of TJ0113 intervention group (Fig. 8B). The levels of ALT and AST were measured to assess the degree of liver function impairment. TJ0113 reduced liver histologic scores compared with the model group. Both ALT and AST levels were significantly elevated in the model mice, whereas the TJ0113 group resulted in lower ALT and AST levels in ALF serum (Fig. 8C–E). Survival analysis showed that viability at 38 h was 0 % in the model group, compared to 10–20 % in the low-dose TJ0113 group (5 mg/kg by gavage and 0.5 mg/kg by intraperitoneal injection) and 40–50 % in the high-dose TJ0113 group (10 mg/kg by gavage and 1 mg/kg by intraperitoneal injection) (Fig. 8F). ELISA of sera from all groups of mice revealed that TAA-induced expression levels of inflammatory factors (IL-6, IL-1β, and TNF-α) were significantly elevated in ALF mice, and the release of inflammatory factors were significantly reduced by TJ0113 pretreatment (Fig. 8G–I). The data indicated that TJ0113 attenuated hepatic tissue injury and inflammatory response in ALF mice, and TJ0113 was not toxic to mice.

### 3.7 TJ0113 inhibits activation of the hepatocyte apoptosis and the NF-kB/NLRP3 pathway

**Fig. 9:**
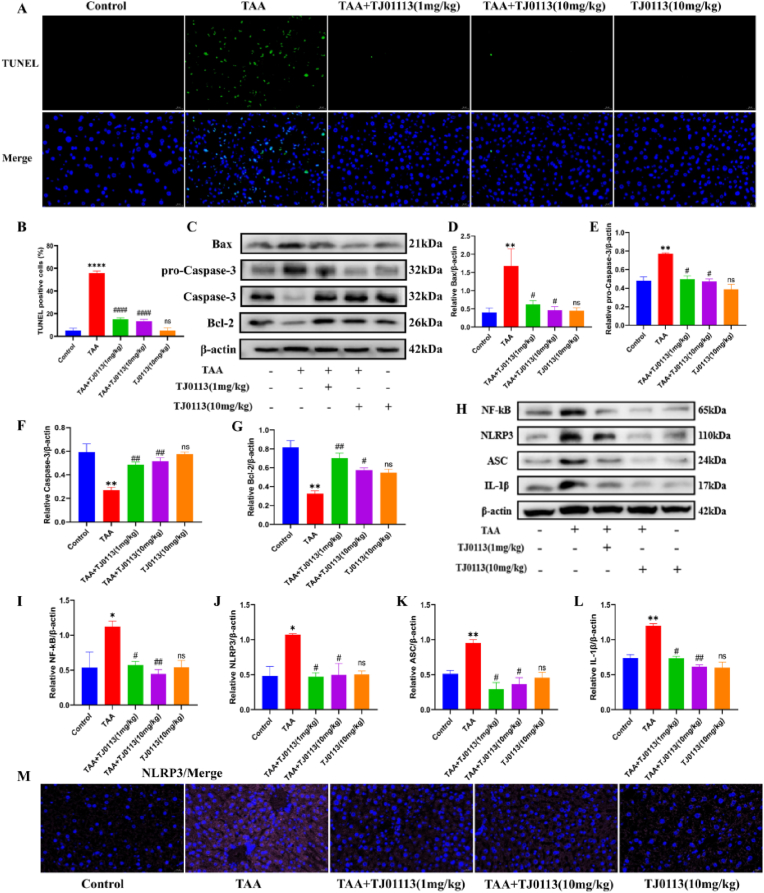
TJ0113 ameliorates massive apoptosis and activation of the NF-kB/NLRP3 pathway in hepatocytes from **ALF mice.** (A-B)TUNEL staining was used to detect the number of apoptotic cells in liver tissue sections and the percentage apoptosis score of hepatocytes. (C) Representative immunoblotting images and quantification of Bax (D), pro-Caspase-3 (E), Caspase-3 (F) and Bcl-2 (G). (H) Representative immunoblotting images and quantification of NF-kB (I), NLRP3 (J), ASC (K) and IL-1β (L) in liver tissue. (M) Immunofluorescence images of NLRP3. Compared with the control group, ∗p < 0.05, ∗∗p < 0.01, ∗∗∗∗p < 0.0001. Compared with the TAA group, #p < 0.05, ##p < 0.01, ####p < 0.0001; ns, not significant. Data are expressed as mean ± SD.

Hepatocyte apoptosis is a key factor leading to acute liver injury. To investigate the effect of TJ0113 on TAA-induced changes in hepatocyte apoptosis. We used different methods to detect the changes of apoptosis key proteins. The increase of apoptotic cells in the model group was seen in the TUNEL assay, and the pretreatment with TJ0113 decreased the percentage of apoptotic cells (Fig. 9A–B). Western blot was used to detect the expression of related proteins, and the expression of anti-apoptotic proteins Bcl-2 and Caspase-3 was down-regulated, and pro-apoptotic proteins (pro-Caspase-3 and Bax) were elevated in the model group. However, compared with the model group, TJ0113 pretreatment could partially reverse the onset of apoptosis by regulating key proteins of the internal and external apoptotic pathways (Bcl-2, Bax, pro-Caspase-3, Caspase-3) (Fig. 9C–G). These data suggest that TJ0113 reduces TAA-induced apoptosis in liver cells of ALF mice. We examined key molecules of the NF-kB/NLRP3 inflammatory pathway, and protein expression of NF-kB, NLRP3, ASC, and IL-1β were significantly elevated in the TAA group compared with the control group, a change that was suppressed by TJ0113 (Fig. 9H–L). In addition, we found that in TAA-induced ALF mice, NLRP3 inflammatory vesicles were massively activated and released large amounts of inflammatory factors. After TJ0113 treatment, the activation of NLRP3 inflammatory vesicles was effectively inhibited (Fig. 9M). In conclusion, TJ0113 alleviated massive hepatocyte apoptosis and inflammatory response in ALF mice.

## 4 Discussion

In this study, we used a combination of basic research and Raman spectroscopy together to investigate the role of mitophagy in ALF and the novel mitophagy inducer TJ0113 can induce mitophagy to improve ALF. Raman imaging revealed that TAA-induced ALF mice exhibited a marked change in the 1128 cm^−1^ Raman peak—indicative of mitochondrial cytochrome redox imbalance—compared to controls. Treatment with TJ0113 restored the 1128 cm^−1^ signal, as confirmed by normalization to the stable 1008 cm^−1^ peak. These findings provide direct, real-time evidence that TJ0113-induced mitophagy effectively mitigates mitochondrial dysfunction in ALF. *In vivo* experiments, the administration of TJ0113to TAA-induced ALF mice reduced mitochondrial damage and restored mitochondrial homeostasis. TJ0113 induced mitophagy by targeting Mcl-1 via the PINK1/Parkin and ATG5 pathways, and then acted on LC3A. TJ0113 mainly affected the lipid metabolism and nucleotide metabolism of the liver in ALF mice. RNA-seq showed that TJ0113 treatment reduced apoptosis and inflammation in ALF mice by modulating the PI3K/AKT signaling pathway. TJ0113 treatment attenuated hepatic function, pathological tissue changes, and survival in ALF mice.

The pathogenesis of ALF involves multiple stages, including abnormal lipid accumulation and lipid peroxidation, uncontrolled aseptic inflammation, and massive hepatocyte death [34]. In addition to this, there is growing evidence that mitochondrial dysfunction is associated with the development and progression of ALF [35,36]. Mcl-1 is a newly discovered mitophagy receptor [37]. Mcl-1 dissociates from the pro-apoptotic protein Bax. Fragmented Mcl-1 enhances interaction with LC3A and thus promotes mitophagy [19]. In ALF, elevated Mcl-1 and LC3 expression in hepatocytes antagonizes apoptosis as well as inducing mitophagy in the face of severe stress and injury [38]. UMI-77 can induce mitophagy to ameliorate Alzheimer's disease, renal fibrosis, premature aging syndrome and sepsis [[20], [21], [22], [23]]. In our study, we obtained TJ0113 from the Liangzhu Research Center. TJ0113 has better drug-forming properties and stronger selective autophagy induction than UMI -77, and TJ0113 has been approved by the FDA to enter clinical studies. We used TJ0113 to treat TAA-induced ALF mice and found that mitophagy markers (LC3A/B, Mcl-1, and LAMP1) were elevated while TOM20 was decreased. Subsequently, we examined the expression of molecules related to the classical and non-classical pathways of mitophagy. The results show that TJ0113 can induce mitophagy through PINK1/Parkin and ATG5 signaling. In addition, we found increased 8-OHDG expression in ALF mice, indicating severe oxidative DNA damage. Electron microscopy experiments showed that mitochondria exhibited irregular swelling, length shortening and cristae breakage after TAA treatment. Imbalance in mitochondrial dynamics is closely associated with ALF, viral hepatitis, and non-alcoholic fatty liver disease non-alcoholic fatty liver disease (NAFLD) [8,39]. Mitochondria are highly dynamic organelles, regulated by fusion and division processes. These morphological changes coincide with changes in the expression of markers associated with mitochondrial dynamics (e.g. Drp1, Opa1, and Mfn2), suggesting that mitochondria tend to divide. Treatment of TJ0113 improves mitochondrial adaptation. In conclusion, TJ0113 induces mitophagy to improve mitochondrial adaptation and improves survival, liver function and histopathological changes in ALF mice.

We also resorted to histological analysis to further explore the specific mechanism by which TJ0113 induces mitophagy to ameliorate ALF. We not only performed hepatic metabolomics studies, but also analyzed hepatic gene expression by RNA-seq in mice in the TJ0113 treatment group. The results of metabolomics analysis showed that the differential metabolites between the TAA and TAA + TJ0113 groups were dominated by lipids and lipid-like substances. KEGG enrichment analysis revealed that these metabolites were mainly enriched in lipid metabolism. Lipid metabolism has been found to play an important role in ALF [40,41]. The mitochondrion is an organelle that plays an important role in regulating redox, lipid metabolism and cell death in hepatocytes [8]. TJ0113 can regulate ALF progression by modulating lipid metabolism in liver tissues. We used RNA-seq to further explore the alteration of liver genes in TAA-induced ALF mice by TJ0113. We concluded by RNA-sequencing that TJ0113-induced activation of mitophagy regulates the PI3K/AKT signaling pathway and thus inflammation and apoptosis in ALF mice in an *in vivo* model. Dysregulated activation of the PI3K/AKT signaling pathway is thought to be one of the major drivers of ALF progression [42,43]. Sustained activation of the PI3K/AKT pathway increases oxidative stress and enhances the expression of proinflammatory factors, leading to hepatocyte inflammation and apoptosis [44]. More and more studies have found that mitophagy is closely related to the P13K/AKT pathway. Li J.et al. found that JianPi LiShi YangGan formula (YGF) could ameliorate ALF by promoting mitophagy through inhibiting the PI3K/AKT/mTOR pathway [45]. In addition to the traditional molecular biological detection means, interestingly, Raman spectroscopy was used in this study for detection, realizing the synergistic use of optics and medicine.

Raman spectroscopy is increasingly being used in medicine, including in the diagnostic identification of diseases and the detection of dynamics [46,47]. This approach has the advantages of being fast, continuous, non-destructive and non-marking compared to traditional detection methods [28]. Previous studies have shown that patients with ALF with mitochondrial injury have a poor prognosis and a high mortality rate [8,38,48]. Similar results were observed in the present study, where Raman imaging-based mitochondrial redox state revealed ALF outcomes including inflammation, apoptosis and ALF mortality. Mitochondrial cytochromes can represent the mitochondrial redox state that carries out electron transfer in the electron transport chain [49,50]. The point-scan Raman imaging system can accurately identify characteristic peaks at 750 cm^−1^, 1128 cm^−1^, and 1585 cm^−1^ that correspond to vibrations of porphyrins, Cb-CH3 side radicals, and symmetric vibrations of methyl bridges (CaCm, CaCmH bonds) [51]. They are all sensitive to the redox state of cytochromes and are involved in cytochrome (cyt) c and cyt *b* [52,53]. In this study, considering the weak Raman signal at 750 cm^−1^ and the potential interference at 1585 cm^−1^, we finally used the normalized peak at 1128 cm^−1^ as the Raman detection index for mitochondrial damage in mouse blood. Considering the complexity of the Raman signal in blood, we chose the phenylalanine peak at 1008 cm^−1^ as the normalized standard because of its relative stability in different samples, which represents a stable conformational change of the protein, thus normalizing the spectrum. To date, measurement of mitochondrial dysfunction in ALF has relied on tissue biopsies using common biochemical tools, which can damage mitochondria *in vivo* [54]. Using Raman technology to monitor mitochondrial dysfunction has made tremendous strides, including in sepsis, diabetes, and cancer [51,52,[55], [56], [57]]. We removed liver tissues from mice at 24 h when the mitochondrial damage was most severe for Raman signal detection. The assay results revealed that the Raman signals of ALF mice were significantly different from those of the control and TJ0113 treatment groups. Similarly, we extracted mitochondria from liver tissue for Raman detection and obtained results consistent with the Raman signal of liver tissue. In addition to Raman testing, we used traditional biochemical and histopathological tests to validate our Raman signal results.

In this study, we chose to use AFLSCRI instead of nonlinear Raman techniques such as Stimulated Raman Scattering (SRS) or Coherent Anti-Stokes Raman Scattering (CARS). Although SRS and CARS have advantages in imaging speed and tissue penetration depth, AFLSCRI can provide complete Raman spectroscopic information at each measurement point, allowing us to conduct comprehensive analysis of multiple biochemical components simultaneously. In addition, the equipment required for AFLSCRI is simpler and easier to obtain, and operates at lower laser power, reducing the risk of light damage to the sample and maintaining cell integrity. Based on the above factors, AFLSCRI is more suitable for our research objectives and helps to accurately evaluate mitochondrial function under experimental conditions. We continuously detected the mitochondrial functional changes after pretreatment with TJ0113. After TJ01113 was given to induce mitophagy 1 h before TAA modeling, the blood of the tail vein of the mice was continuously monitored. The results in Fig. 2 showed that the Raman signal was relatively stable in the 1128 cm^−1^ peak in the control group, whereas it decreased rapidly in the TAA group, and the damage was most serious at the 24 h after modeling (and the mice were dead), which indicated that TAA induced the redox dysfunction of mitochondria, and the content of cytochromes decreased. The Raman signals of the TJ0113-treated groups showed a tendency to increase and then decrease, and reached the peak value at 24 h after the treatment, higher than the control group, and reached the peak value at 30 h after the treatment, which was lower than the control group. These results suggest that TJ0113 can induce mitophagy to reduce mitochondrial damage and thus improve ALF, and similar results have been obtained in the blood of type 2 diabetes mellitus [58].

Sepsis combined with multi-organ failure and cerebral edema remains the leading cause of death in patients with ALF, and early detection and appropriate management can alter the course of ALF [59]. Mitochondrial function, which represents the redox state, has been found to be severely impaired in skeletal and leg muscles in patients with sepsis [60,61]. Raman spectrum measurement was found to be more effective in monitoring gastrocnemius muscle mitochondrial redox status, which can help in early recognition of sepsis severity and prognosis [51]. Therefore, Raman signal detection of mitochondrial redox status in the gastrocnemius muscle may allow early identification and prediction of ALF outcome. We used Raman signaling to continuously monitor the mitochondria of mouse gastrocnemius muscle to assess the therapeutic effect. By evaluating the mitochondrial Raman signals in mouse gastrocnemius muscle after TAA administration 1 h before modeling, 4 h after modeling, and 12 h after modeling, we found that TJ0113 pretreatment and early ALF administration had a therapeutic effect on the progression of ALF. TJ0113 given at 12 h of modeling induced mitophagy with little effect in ALF mice. Raman microscopy has the benefit of being faster, label-free, and portable compared to conventional biochemical tests.

Raman microscopy is increasingly used for a wide range of clinical applications, including term clinical diagnosis, effective intraoperative guidance, and accurate prognosis [28]. The technology is being explored in depth in cancer diagnosis and prediction, providing a new direction for early cancer diagnosis and personalized treatment [62,63]. Compared with traditional clinical methods, micro-Raman has faster and more reliable clinical detection, treatment and monitoring methods to enhance its clinical applications [[64], [65], [66]].

Our study also faces the following limitations: 1) There are various models of ALF, and our study only used one, which imposes certain limitations; 2) We did not perform tests on ALF patients, so future research could consider including this aspect; 3) The skin acts as a barrier to Raman signals from mitochondrial damage in the gastrocnemius muscle. In the future, devices capable of penetrating the skin or topical agents may be developed, but as these have not yet been tested on humans, they present certain limitations. Additionally, spectral overlap among different molecules is an issue that requires attention in Raman spectroscopy analysis [67]. Especially within the complex environment of biological systems, multiple molecules may coexist and generate similar spectral features. In our study, we selected the Raman peak at 1128 cm^−1^ (associated to cytochrome *c*) as an indicator of mitochondrial redox status, mainly due to its clean region with little interference with other mitochondrial molecules. For example, the Gauche C–C stretching (dominant in fluid-phase membranes or unsaturated lipids) vibrations of disordered acyl chains in phospholipids (e.g., mitochondrial membrane lipids) exhibit Raman peaks in the range of 1080–1100 cm^−1^, which is close to but can be distinguished from our targeted Raman signals 1128 cm^−1^; valine has its primary Raman peak at 935 cm^−1^ (the closest secondary peak to 1128 cm^−1^ is 1122 cm^−1^, but still minor interference). Advanced technologies such as SERS (surface-enhanced Raman spectroscopy), polarized Raman spectroscopy (to differentiate ordered (trans) vs. disordered (gauche) lipid phases) and low-temperature Raman (to reduces thermal broadening) can be utilized to reduce potential interference with other mitochondrial molecules. Under the experimental conditions of our study, the Raman peak at 1128 cm^−1^ is shown to be a reliable indicator of mitochondrial redox status for acute liver failure. Finally, while we primarily focused on monitoring cytochrome *c* via Raman spectroscopy to assess mitophagy in ALF, we recognize that incorporating additional biomarkers—such as NADH (with characteristic peaks at 480, 710, and 1600 cm^−1^) and lipid peroxidation products (evidenced by a C=C stretch near 1655 cm^−1^)—could provide a more comprehensive understanding of ALF pathophysiology [68,69]. However, challenges such as spectral overlap and inherently weak signals may require improved Raman methods such as surface-enhanced Raman spectroscopy (SERS) or resonance Raman, or Polarized Raman spectroscopy and further advanced deconvolution approaches (e.g., PCA or PLSR) to improve sensitivity [70]. In future work, we plan to integrate these biomarkers to provide a holistic view of the biochemical changes during ALF, ultimately leading to improved diagnostic and therapeutic strategies.

There are three main innovations presented in this study. First, our use of Raman spectroscopy enables real‐time, label‐free detection of mitochondrial damage in blood. Second, we demonstrate the rapid, non-invasive *in vivo* monitoring of acute liver failure (ALF) progression using Raman imaging. Third, continuous Raman monitoring of the mouse gastrocnemius muscle provides valuable insights that guide drug therapy. Together, these innovations open a new avenue for early clinical diagnosis, therapeutic management, and prognostic evaluation of ALF patients—and may also be applicable to other diseases involving mitochondrial dysfunction. Future studies will be essential to further validate and expand the clinical utility of this Raman approach.

## CRediT authorship contribution statement

**Chunlian Huang:** Writing – review & editing, Writing – original draft, Methodology, Investigation, Formal analysis, Data curation. **Jiaqi Liao:** Writing – original draft, Methodology, Formal analysis, Data curation. **Xufeng Cen:** Writing – review & editing, Resources. **Changwei Jiao:** Resources. **Sijia Chen:** Data curation. **Dong Liu:** Writing – review & editing. **Hang-Shuai Qu:** Writing – review & editing. **Jiansheng Zhu:** Writing – review & editing, Funding acquisition. **Sailing He:** Writing – review & editing, Supervision, Formal analysis, Data curation, Conceptualization.

## Data availability

Data supporting the results of this study are available in the original manuscript and its Supplementary Information. Source data are provided in this article.

## Declaration of competing interest

This manuscript has not been published or presented elsewhere in part or in entirety and is not under consideration by another journal. Animal experiments were approved by the Animal Experimentation Ethics Committee of Taizhou Hospital, Zhejiang Province (Approval No. tzy-2023216). We have read and understood your journal's policies, and we believe that neither the manuscript nor the study violates any of these. There are no conflicts of interest to declare.

## Data Availability

Data will be made available on request.
