# Supplementary material for: TJ0113-induced mitophagy in acute liver failure detected by Raman microspectroscopy
Source: Redox Biol. 2025 Apr 29;83:103654. doi: 10.1016/j.redox.2025.103654 (PMC12098161; doi:10.1016/j.redox.2025.103654)
Supplement: Multimedia component 1 [file mmc1.docx]

**Supplementary information**

**TJ0113-induced mitophagy in acute liver failure detected by Raman microspectroscopy**

**Chunlian Huang^1+^, Jiaqi Liao^2,3,4,+^, Xufeng Cen^5,+^, Changwei Jiao^2^,** **Sijia Chen^1^, Dong Liu^6^, Hang-Shuai Qu^7^, Jiansheng Zhu^1,^* and Sailing He^3,4 ,8,*^**

^1^Department of Infectious Diseases, Taizhou Hospital of Zhejiang Province Affiliated to Wenzhou Medical University, Linhai, Zhejiang, 317000, China.

^2^ Centre for Optical and Electromagnetic Research, College of Optical Science and Engineering, Zhejiang University, Hangzhou, 310058, China

^3^ Zhejiang Engineering Research Center for Intelligent Medical Imaging，Sensing and Non-invasive Rapid Testing, Taizhou Hospital, Zhejiang University, Taizhou, China.

^4^ National Engineering Research Center for Optical Instruments, Zhejiang University, Hangzhou 310058, China

^5^Research Center of Clinical Pharmacy of The First Affiliated Hospital & Liangzhu

Laboratory, Zhejiang University School of Medicine, Hangzhou, China.

^6^Hangzhou PhecdaMed Co., Ltd. third floor, building 2, No.2626. yuhangtang Road, Yuhang District, Hangzhou City, Zhejiang Province, China;

^7^Department of public laboratory, Taizhou Hospital of Zhejiang Province Affiliated to Wenzhou Medical University, Linhai, Zhejiang, 317000, China.

^8^Department of Electromagnetic Engineering, School of Electrical Engineering, Royal Institute of Technology, 100 44 Stockholm, Sweden

+ The equally to this work and are co-first authors or co-first correspondence.

ChunLian Huang:17275833360[@163.com](mailto:huangcl9994@163.com)

Jiaqi Liao:12330095@zju.edu.cn

Xufeng Cen: 0616502@zju.edu.cn

Changwei Jiao:1470791156@qq.com

Sijia Chen: chensijia777@163.com

Dong Liu: [dong.liu@phecdamed.com](mailto:dong.liu@phecdamed.com)

Hang-Shuai Qu: [Quhs9426@enzemed.com](mailto:Quhs9426@enzemed.com)

JianSheng Zhu: [zhujs@enzemed.com](mailto:zhujs@enzemed.com)

Sailing He：sailing@kth.se

* Correspondence:

Sailing He,Department of Electromagnetic Engineering, School of Electrical Engineering, Royal Institute of Technology, 100 44 Stockholm, Sweden. E-mail address: sailing@kth.se (S. He);

Jiansheng Zhu, Department of Infectious Diseases, Taizhou Hospital of Zhejiang Province, Wenzhou Medical University, 150 Ximen Street, Linhai 317000, Zhejiang Province, China. E-mail: zhujs@enzemed.com.

**Table of contents**

Supplementary Fig. 1:Raw Raman spectra (without baseline correction) and the extracted baseline extraction at two different wavenumber ranges.

Supplementary Fig. 2: Raw Raman spectra and Raman signal with fluorescence baseline removed at two different wavenumber ranges.

Supplementary Fig. 3: Experimental and simulated spectra used to demonstrate the efficiency of ALS calibration methods


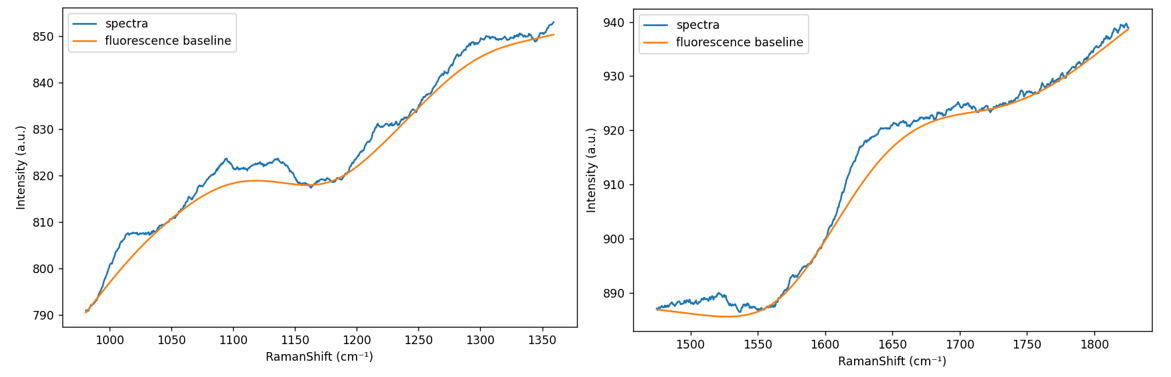


Supplementary Figure 1. Raw Raman spectra (without baseline correction) and the extracted baseline extraction at two different wavenumber ranges.

In Figure S1, we present a raw Raman spectrum along with the fluorescence baseline extracted via our ALS algorithm. This figure clearly shows that the baseline is smoothly varying.


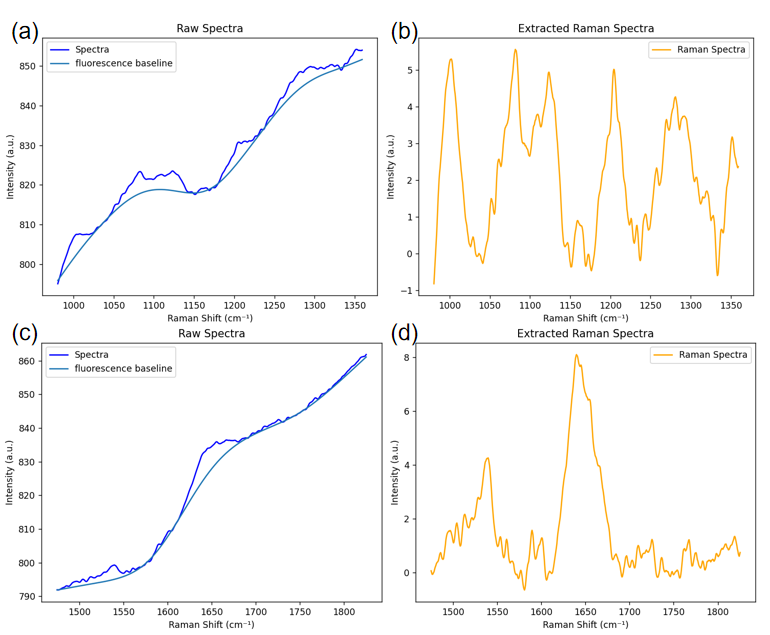


Supplementary Figure 2. Raw Raman spectra and Raman signal with fluorescence baseline removed at two different wavenumber ranges.(a) and (c) Raw Raman spectra, (b) and (d) Raman signal with fluorescence baseline removed.

Figure S2 includes the same representative spectrum after SG (Savitzky-Golay) filtering, which are the same as Figure S1. It shows both the uncorrected and baseline-corrected Raman signals.

By comparing Figure S2(a) and (b), Figure S2(c) and S2(d), it can be observed that Subtracting the baseline via the ALS method enhances the resolution of the Raman peaks.


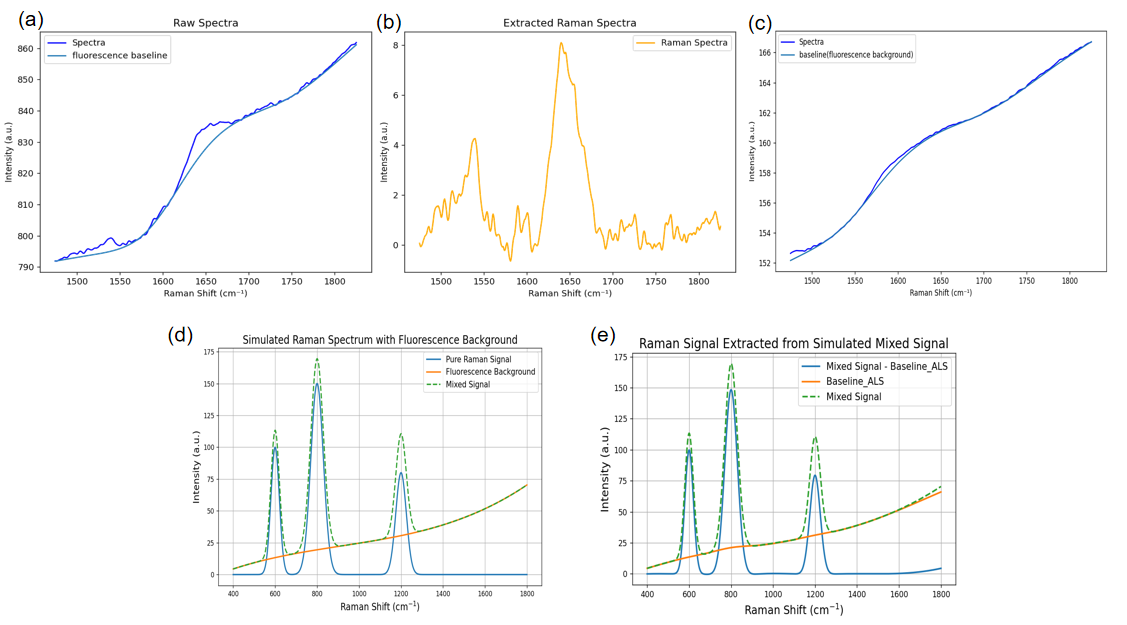


Supplementary Figure 3. experimental and simulated spectra used to demonstrate the efficiency of ALS calibration methods. (a) The original experimental spectrum together with the fluorescence baseline extracted by our ALS algorithm. (b) The Raman spectrum obtained after subtracting the extracted fluorescence baseline from the original signal. (c) (d) The simulated pure Raman signal and the fluorescence background.. (e) The spectrum of the sample and the extracted fluorescence baseline, which is nearly the same as the original spectra (as expected).

Figure S3 (a) and (b) show the original experimental spectrum with and without fluorescence baseline. By reducing the laser intensity irradiated on the sample, we obtained sample data with nearly zero Raman signal (i.e., (fluorescence-only). However, the corresponding fluorescence signals are also weaker but have similar shapes (Figure S3(c)).

Due to the inherent complexity of biological samples, it is challenging to obtain a negative control specimen that exhibits the same fluorescence characteristics as our experimental samples while being completely devoid of Raman activity. Therefore, to further address this issue and validate the reliability of our ALS correction method, we have also designed a simulation experiment. In this simulation, we generated a synthetic Raman spectrum by summing multiple Gaussian peaks and created a fluorescence background using a cubic polynomial function; these were then combined to form a mixed spectrum (of Raman and fluorescence spectra). By applying the ALS algorithm, we demonstrate that the extracted Raman signal closely matches the original Raman input, thereby supporting the efficiency of the present correction method.
